# Supplementary material for: Pharmacogenomic Profile and Adverse Drug Reactions in a Prospective Therapeutic Cohort of Chagas Disease Patients Treated with Benznidazole
Source: Int J Mol Sci. 2021 Feb 16;22(4):1960. doi: 10.3390/ijms22041960 (PMC7920452; doi:10.3390/ijms22041960)
Supplement: Supplementary file 1 [file ijms-22-01960-s001.pdf]

Supplementary Material

Author Contributions: Conceptualization, methodology, Investigation, formal analysis and writing – original draft **Lucas AM Franco**, Conceptualization, Investigation, formal analysis and writing – original draft, **Carlos HV Moreira**, formal analysis, data curation, writing -review and editing, **Lewis F Buss**, project administration and supervision, **Lea C Oliveira**, data curation and writing -review and editing, **Roberta CR Martins**, project administration, **Erika Manuli**, investigation, **José AL Lindoso**, validation and data curation, **Michael P Busch**, software, formal analysis, resources and writing - review and editing, **Alexandre C Pereira**, Conceptualization, Resources, Visualization, funding acquisition and supervision **Ester C Sabino**. All authors have read and agreed to the published version of the manuscript.

Funding: This work was supported by the Bill and Melinda Gates foundation under grant [nº OPP1071295/Subagreement 7636], the Conselho Nacional de Desenvolvimento Científico e Tecnológico (CNPq) [nº 467043/2014-0], the Coordenação de Aperfeiçoamento de Pessoal de Nível Superior (CAPES) - [LAMF] and Mobilidade Santander project [PRPG - 03/2018] – [LAMF].

Conflict of interest: All authors declare no conflict.

Table S1: Significant DEGs from transcriptomic assay for the comparison T0dxT60d by each group, gNADR and gADR:

**11 significant genes from the comparison T0dxT60d in the gNADR group**		
**Gene**	**logFC**	**P Value**
**CHAD**	-1.41622	0.001336
**COL18A1**	-1.61027	0.001807
**HRK**	-1.29777	0.04754
**IFI44L**	1.856523	0.002944
**IFIT1**	1.339136	0.017201
**N4BP3**	-1.43522	0.022686

**OAS3**	1.472554	0.001609
**RSAD2**	1.6265	0.018014
**SERPING1**	1.330768	0.005578
**TNFRSF17**	-1.37666	0.001339
**ZNF365**	-3.40626	0.042609
**173 significant genes from the comparison T0dxT60d in the gADR group**		
**Gene**	**logFC**	**P Value**
**ACTC1**	2.45055	0.021523
**ADGRA3**	1.837984	0.00172
**ADRA2A**	2.213965	0.005612
**ALOX15B**	1.63046	0.001612
**APCS**	2.623452	0.028767
**APLP1**	1.395323	0.039663
**APOA4**	2.583216	0.036837
**APOB**	2.401228	0.038535
**ARC**	1.818692	0.005489
**ARHGEF17**	1.364797	0.005939
**ASPM**	1.631608	0.000936
**ASS1**	1.359996	0.009166
**BHLHA15**	1.447433	0.013638
**C10orf55**	1.785034	0.034693
**CACNA1D**	2.131394	0.000182
**CALML5**	1.558004	0.03891
**CAMSAP3**	1.332978	0.029195
**CATSPER1**	1.854484	0.038522
**CCL18**	1.57277	0.032925
**CCL19**	1.740797	0.022269
**CCL21**	1.539176	0.03734

**CCL26**	1.886223	0.042051
**CCL27**	1.597928	0.0195
**CCNE1**	1.785039	0.000134
**CD1B**	1.830666	0.0000249
**CDH3**	1.844506	0.002509
**CH25H**	1.412112	0.04542
**CHEK1**	1.624458	0.027361
**CLDN16**	1.760372	0.022516
**CLDN3**	1.494903	0.016497
**CLEC4M**	2.171416	0.031127
**CLSTN2**	2.139265	0.034356
**CMA1**	1.797232	0.026788
**CNTFR**	1.894085	0.046008
**COL4A1**	2.291074	0.005858
**COMP**	1.849264	0.03924
**CREB3L1**	1.945041	0.012901
**CXCL6**	1.381453	0.021078
**CYP11B1**	2.399168	0.005688
**CYP24A1**	2.597834	0.004537
**CYP2B6**	1.878145	0.047007
**CYR61**	2.139308	0.030631
**DAB2IP**	1.468407	0.0179
**DACT3**	1.601558	0.04153
**DEFA5**	1.964974	0.02423
**DEPDC1**	1.661597	0.008962
**DHRS2**	1.662547	0.045173
**DIO3**	1.777374	0.021156
**DIRAS3**	2.733008	0.004499
**DLL3**	1.37263	0.013708

**DPPA3**	1.495257	0.044376
**DRD5**	1.298153	0.046907
**ELF3**	1.93934	0.000998
**EMP1**	1.466612	0.000676
**ERBB3**	1.753003	0.008316
**FBXL2**	2.26805	0.0204
**FGFR4**	1.364339	0.013233
**FZD8**	1.292147	0.032343
**GABRB1**	3.017873	0.024332
**GATA6**	2.131049	0.007952
**GLYATL1**	2.039689	0.035696
**GNB3**	1.661934	0.003563
**GOLGA6B**	1.778258	0.010695
**GOLGA6C**	1.720963	0.032767
**GPC5**	2.590636	0.034321
**GPHA2**	1.732325	0.008103
**GPR32**	1.621702	0.018819
**GSTA1**	2.68789	0.002947
**GSTA3**	3.599436	0.002009
**HIST1H2BJ**	1.510803	0.003287
**HPN**	1.556879	0.018036
**HPX**	1.336865	0.029297
**HSD11B2**	1.475301	0.005652
**HSPB6**	1.504065	0.023123
**IFNA6**	1.50955	0.047139
**IFNL1**	2.044792	0.02705
**IFNL2**	2.139206	0.033005
**IFNW1**	1.407464	0.030428
**IL11**	1.531422	0.024418

**IL19**	2.517126	0.004685
**IL20**	2.415008	0.017823
**IL22**	1.647637	0.003771
**IL24**	1.840818	0.012408
**IL26**	3.43641	0.009454
**IQGAP3**	1.500754	0.004505
**JUN**	1.664272	0.017361
**KCNJ11**	1.565567	0.028871
**KIF20A**	1.258003	0.008607
**KLK3**	1.741423	0.002557
**KLK5**	2.05524	0.034665
**KRT10**	1.840699	0.01756
**KRT14**	1.964658	0.016522
**KRT16**	1.995597	0.039191
**KRT6B**	2.519766	0.025605
**LRRC34**	2.448772	0.002658
**MAB21L1**	1.316935	0.028695
**MAFA**	1.536789	0.024843
**MAP1LC3C**	2.416966	0.031011
**MARK1**	2.319701	0.007639
**MC3R**	1.773516	0.020588
**MCAM**	1.832275	0.005964
**MFAP4**	1.461485	0.003478
**MIOX**	1.876908	0.005819
**MIR31HG**	2.608996	0.001883
**MIR548I1**	1.440914	0.004891
**MIR548I2**	1.507455	0.006838
**MIR548I3**	1.59069	0.013955
**MLC1**	1.924571	0.001525

**MMD2**	2.266528	0.031468
**MMRN2**	1.650436	0.042361
**MOS**	1.600695	0.033216
**MRC2**	1.569296	0.003275
**MRGPRX1**	1.45416	0.048126
**MST1R**	1.658162	0.009645
**MT3**	1.571337	0.007037
**MUC5B**	1.750745	0.012175
**MYH6**	1.778377	0.031352
**MYRF**	1.606156	0.011183
**NCR2**	2.145459	0.027939
**NOG**	2.780361	0.00031
**NOTCH3**	1.563561	0.006884
**NOTCH4**	2.019469	0.03974
**NPPA**	1.582863	0.010828
**NUPR1**	1.783574	0.01851
**NXF3**	1.480634	0.025508
**NYNRIN**	1.587131	0.011739
**P4HA3**	1.670191	0.026239
**PACSIN3**	1.64087	0.04635
**PAEP**	1.742416	0.034446
**PDGFRA**	2.035476	0.044884
**PKLR**	2.38023	0.009216
**PKP3**	1.807048	0.01851
**PLA2G2A**	2.734578	0.016239
**PLK2**	1.355021	0.004875
**PLOD2**	1.86641	0.000147
**PMEL**	1.823925	0.002291
**PPP1R1B**	1.273802	0.03015

**PRKAG3**	2.385151	0.024401
**PRPH**	1.784126	0.029832
**RAC3**	1.308664	0.008868
**RAMP2**	2.127556	0.011208
**RGS4**	1.932406	0.044453
**RHBDF1**	1.886511	0.004597
**RHCG**	2.391264	0.027449
**RXFP3**	1.478782	0.031246
**RYR1**	2.266577	0.003275
**SERPINE1**	1.497074	0.000972
**SHBG**	1.846593	0.035275
**SLC52A1**	1.439858	0.007942
**SLC5A2**	1.374723	0.016504
**SLCO2A1**	1.629517	0.024547
**SLCO4A1**	1.49533	0.009903
**SNX7**	1.326715	0.022127
**SOX11**	1.450997	0.040795
**SOX2**	1.704571	0.039581
**STAP2**	1.454356	0.002956
**STRA6**	2.880188	0.019315
**TACSTD2**	1.485803	0.026471
**TAF7L**	3.095862	0.004627
**TBX6**	1.318594	0.002841
**TEAD3**	1.483095	0.016398
**TEAD4**	2.330419	0.011743
**TGFB2**	1.480613	0.007428
**TIE1**	2.035245	0.004696
**TKTL2**	1.442721	0.038432
**TPSD1**	1.795225	0.03743

**TRIL**	1.558699	0.026059
**TSPAN1**	1.977775	0.013676
**TYRO3**	1.788688	0.027913
**UGT1A10**	1.755202	0.019476
**XAGE2**	2.257155	0.018549
**ZNF668**	1.574247	0.028353
**ZNF750**	2.040499	0.010593

Table S2: Signaling pathways activated from significant genes expression by groups. Genes in bold on the Molecules in Network column were dosed, table S1, and considered significant. The Score value is the power of the association of the pathway with the Top Diseases and Functions ranked by the literature that corroborates these findings. The Top diseases and Functions column presents in bold the clinical conditions observed or expected in patients from each of the groups, gNADR and gADR:

**gNADR patients**			
**Molecules in Network**	**Score**	**Focus Molecules**	**Top Diseases and Functions**
AGER, BTK**, CHAD, COL18A1**, DDIT4, DDX58, EPHB2, ERK1/2, F11, **HRK**, HTRA1, ID3**, IFI44L**, IFIH1, **IFIT1**, IFN type 1, IFNG, Interferon alpha, IRF7, JUNB, LBP, NRP1, **OAS3**, P38 MAPK, p70 S6k, **RSAD2**, SELP, **SERPING1**, STAT1, STAT2, TCR, TNF, **TNFRSF17**, TNFSF13B, **ZNF365**	24	10	**Antimicrobial Response, Inflammatory Response, Organismal Survival**
**N4BP3**,YWHAG	3	1	Cell Morphology, Cell-To-Cell Signaling and Interaction, Cellular Assembly and Organization

**gADR patients**			
**Molecules in Network**	**Score**	**Focus Molecules**	**Top Diseases and Functions**

Ap1**, CALML5, CCL21,** Cg**, CLSTN2, CXCL6, CYR61, ELF3, EMP1, FZD8, IFNL1**, Iga, IgG**, IL11, IL19, IL20, IL22, IL24, IL26,** IL12 (complex)**, JUN, KRT10, KRT16, MCAM, NCR2,** NFkB (complex)**, RGS4,** Secretase gamma**, SHBG, SLC52A1, STAP2, TACSTD2, TIE1, TPSD1, TYRO3**	42	28	**Dermatological Diseases and Conditions**, **Organismal Injury and Abnormalities**, Cellular Movement
**ADRA2A,** Akt**, CCL19, CCL26, CCL27, CCNE1, COL4A1, CYP24A1, DAB2IP, DEFA5, DLL3, ERBB3,** ERK, ERK1/2**, FGFR4, GATA6, GOLGA6A (includes others)**, Histone h3, **KLK3, KLK5, KRT14, KRT6B, MRGPRX1,** Notch**, NOTCH3,** P38 MAPK**, PDGFRA,** PI3K (family)**, PLOD2, PPP1R1B, RXFP3, SERPINE1, SOX2,** TCF**, TGFB2**	39	27	Cancer, **Gastrointestinal Disease**, **Organismal Injury and Abnormalities**
ANGPTL3, APOA1, **APOA4, APOB**, APOC2, **ARHGEF17**, CCR5, CDC42EP4, CETP, **CH25H**, CXCL8, CYP11A1, DAB2, FANCC, FSH, **GPC5**, GPLD1, HMOX1, **HPX, HSPB6, IFNL2**, KAT5, KRT18, LDLR, Lh, **MT3, MYH6, MYRF**, NLK, Rac, **RGS4, SNX7, TKTL2,** TLR4, **TYRO3**	16	15	Lipid Metabolism, Small Molecule Biochemistry, Vitamin and Mineral Metabolism
**ADGRA3, ALOX15B**, AR, **ASPM,** ATF1**, CCL18, COMP**, CXCL5, **CYP11B1**, CYP17A1, **DEPDC1**, FOXO1, **GSTA1**, GSTA2, **GSTA3**, IGHE, IL13, IL17D, IL7R, JAG1, JAK1, **MIOX**, **MMRN2, MUC5B**, NPPB, NR5A1, PIAS1, SAA1, SREBF1, **TEAD3**, THBS1, TNF, UBE2I, WNT5A, **ZNF750**	15	14	Lipid Metabolism, Small Molecule Biochemistry, Vitamin and Mineral Metabolism
AGO2, AREG, BIRC5, **C10orf55**, CRYAB, DLG1, DNAJB6, ENG, FLOT2, **FZD8**, HEY1, **IQGAP3, mir-548**, miR-16-5p(and other miRNAs w/seed AGCAGCA), miR-34a-5p(and other miRNAs w/seed GGCAGUG), **MOS, MRC2, NYNRIN**, PLAU, **PLK2, PRKAG3**, RB1CC1, S100A2, **SERPINE1**, **SLC5A2**, SMAD3, SNAI2, TCF7L2, **TGFB2**, TP53, TP73, **TSPAN1**, WT1, WWOX, **ZNF668**	15	14	Cardiovascular System Development and Function, Organismal Development, Cellular Development
**ARC, CACNA1D, CAMSAP3**, CCND1, CEBPA, **CLDN3, CLDN16**, CXCL9, CXCL11, **CYP2B6, CYR61**, DNM1L, ETV5, GLI1, GLI2, **GLYATL1**, GPER1, HES1, Mek,mir-515, MYB, NFE2L2, OLR1, **PKP3, PMEL, PRPH**, RBP1, RELA, RXRA, **STRA6, TEAD4**, TP63, **UGT1A7 (includes others)**, VDR, VEGFC	15	14	Cellular Growth and Proliferation, Tissue Development, Cellular Development

ACTA2, **APLP1,** APP, **ASS1**, BMP7, CD44, **CMA1**, CTGF, **DIO3, FBXL2**, FGF2, FN1, GATA4, **GPR32**, HAND2, HMOX1, HSPA8, IL6, KAT5, **MFAP4**, MIF, MYOC, MYOCD, NEUROG1, **NOG**, NOS2, **NPPA, PLA2G2A**, RAF1, **RHBDF1**, SMPD2, **SOX11, TAF7L**, TBX5, THBS1	13	13	Cellular Development, Embryonic Development, Organismal Development
AGRP, BMP4, CD274, CEACAM1, **CHEK1, CREB3L1, DIRAS3, DRD5**, ERVW-1, GAB2, GNA13, HEY1, **HSD11B2**, IL2, **IL22**, IL6R, JAK1, LEP, **MARK1, MC3R, NOTCH4, NUPR1, NXF3, PAEP**, POMC, PPARD, Ppp2c, SMOOTH MUSCLE ACTIN, STAT3, VEGFA, WT1, **XAGE2**, XPO1, YWHAG, YWHAH	13	13	Tissue Development, Cellular Development, Cellular Growth and Proliferation
**BHLHA15**, CCL8, **CCL18, CD1B, CDH3**, CEBPB, **CNTFR**, CSF2, CSF1R, CXCL5, EP300, FNDC3A, HNF4A, IL1A, IL1B, ITGB8, **JUN**, JUND, MAP2K7, mir-146, **MIR31HG**, MLXIPL, MMP10, **MST1R**, NFKBIZ, NR4A2, **PKLR,** PTX3, **RHCG, RYR1**, SAA1, SFTPD, **SLCO4A1**, SOD2, TREM1	12	12	Cellular Function and Maintenance, Cellular Movement, Inflammatory Response
**APCS**, BTK, CCL3, CCL4, **CLEC4M**, CRP, CXCL5, CXCL9, CXCL11, DRAP1, GCG, **GNB3, HIST1H2BJ**, HNF1A, HNF4A, IFIT1, **IFNA6, IFNL1, IFNW1**, IGF1, IL15, INS, Interferon alpha, IRF9, **KCNJ11, KIF20A,** MX1, PI3K (complex), **RAC3**, RARB, STAT2, TCF7L2, TLR3, TNFSF10, **TRIL**	10	11	Cell-To-Cell Signaling and Interaction, **Hematological System Development and Function**, Immune Cell Trafficking
**ACTC1**, ADM, CALCRL, **DHRS2, DPPA3**, EOMES, NANOG, POU5F1, **RAMP2**, Smad2/3, **SOX2**, SOX17, **TBX6**	7	6	Cellular Function and Maintenance, Cell-To-Cell Signaling and Interaction, Cellular Development
DNMT3B, **LRRC34**	1	1	DNA Replication, Recombination, and Repair, Developmental Disorder, **Gastrointestinal Disease**
**MAFA**, PDX1	1	1	Cellular Development, Cellular Growth and Proliferation, **Digestive System Development and Function**
MMP14, **SLCO2A1**	1	1	Connective Tissue Disorders, **Dermatological Diseases and Conditions**, Drug Metabolism

HOXA13, **MAB21L1**	1	1	Embryonic Development, Organ Development, Organ Morphology
**MLC1**, MYF6	1	1	Developmental Disorder, Hereditary Disorder, **Neurological Disease**
**MAP1LC3C,** PLEKHM1	1	1	Connective Tissue Disorders, Developmental Disorder, Hereditary Disorder
CACNA1I, **CATSPER1**	1	1	Developmental Disorder, Endocrine System Disorders, Hereditary Disorder
KRAS, **MMD2**	1	1	Cancer, Cardiovascular Disease, Cell Cycle
**DACT3**, mir-31	1	1	Embryonic Development, **Hair and Skin Development and Function**, Organ Development
**P4HA3,** P4HB	1	1	Connective Tissue Disorders, Developmental Disorder, Hereditary Disorder
**GABRB1,** GABRG2	1	1	Infectious Diseases, **Neurological Disease**, Organismal Injury and Abnormalities
**GPHA2,** GPHB5	1	1	Cell Signaling, Nucleic Acid Metabolism, Small Molecule Biochemistry
**HPN**, SPINT1	1	1	Immunological Disease, Organismal Injury and Abnormalities, Cancer
ADAM12, **PACSIN3**	1	1	Cancer, Connective Tissue Development and Function, **Hematological Disease**

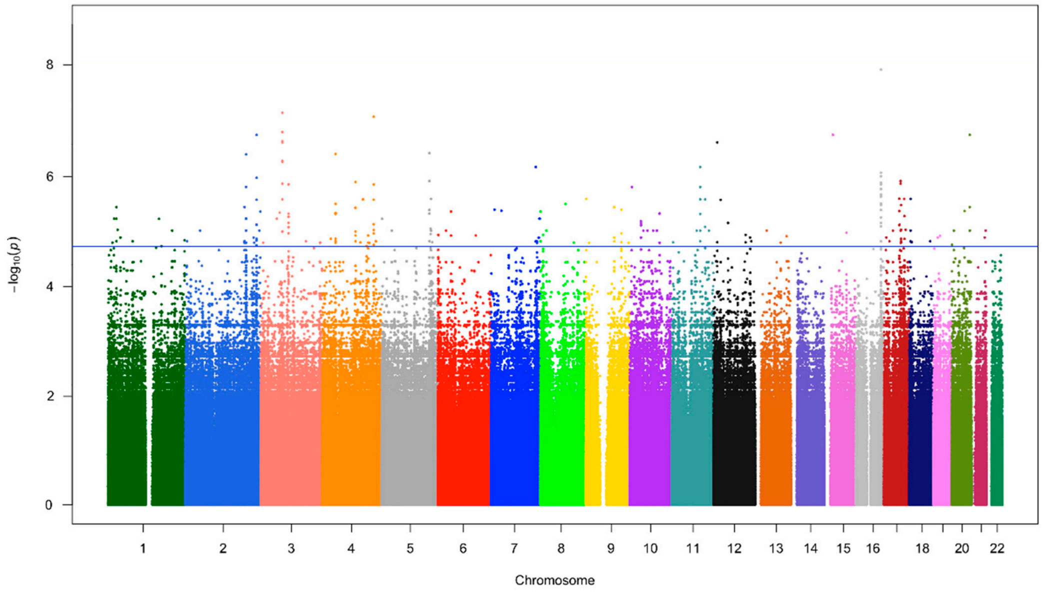

Figure S1: Manhattan plot: All SNPs, from GWAS results, were distributed by the chromosomes they are located and classified by P Value. The blue line represents the cut-off line at 10-5, P value SNPs beneath that were classified as indicative of significance.

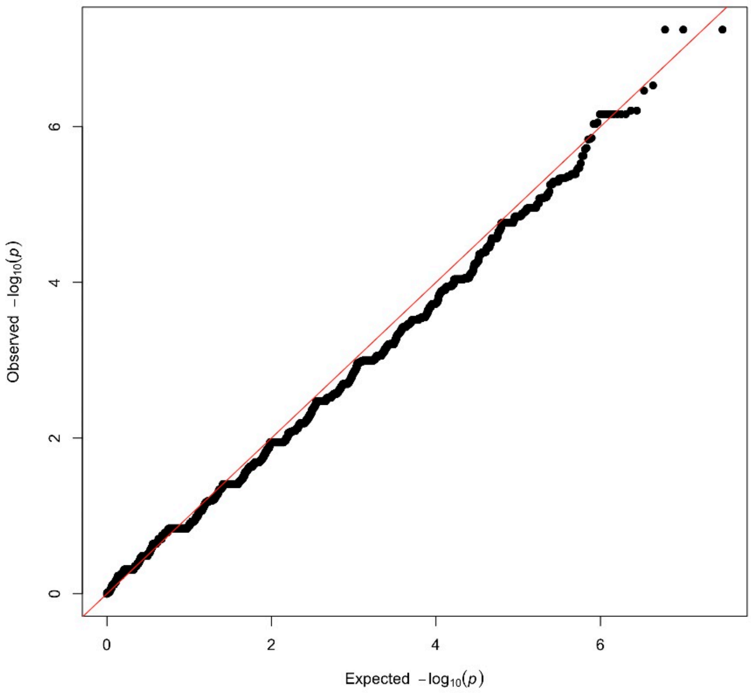

Figure S2: Quantile-quantile plot: The p values observed for each SNP were plotted in ascending order, -log10 (p), against the expected p values, in a Chi-square distribution sample.
